# Assessing the effects of virtual reality-based positive psychotherapy on emotion, life satisfaction, and suicidal ideation in major depression: A mixed-methods randomized controlled trial

**DOI:** 10.1371/journal.pone.0354610

**Published:** 2026-07-30

**Authors:** Fahimeh Alsadat Hosseini, Kimia Bahman, Maryam Shaygan, Shahrazad Yektatalab

**Affiliations:** 1 Community Based Psychiatric Care Research Center, School of Nursing and Midwifery, Shiraz University of Medical Sciences, Shiraz, Iran; 2 Student Research Committee, School of Nursing and Midwifery, Shiraz University of Medical Sciences, Shiraz, Iran; University of Pennsylvania Perelman School of Medicine, UNITED STATES OF AMERICA

## Abstract

**Background:**

Positive psychotherapy (PPT) targets the enhancement of well‑being in individuals with major depressive disorder (MDD), and virtual reality (VR) offers an immersive medium for delivering such experiential interventions. However, most VR-based approaches for depression are grounded in cognitive‑behavioral frameworks, leaving the application of PPT within VR environments largely unexplored. This study examined the effects of VR-based PPT on emotional experiences, life satisfaction, and suicidal ideation in individuals with MDD.

**Methods:**

In this sequential explanatory mixed‑methods study, 78 patients were randomly assigned to VR-based positive psychotherapy (intervention; n = 39) or an equivalent face-to-face format (control; n = 39). Both groups received three sessions of positive psychotherapy, differing only in delivery mode. The quantitative phase assessed changes in affect, life satisfaction, and suicidal cognitions before and after each session using the PANAS, SWLS, and B-SCS. A subsequent qualitative analysis of the intervention group was conducted to contextualize and further interpret the quantitative findings.

**Results:**

The results demonstrated a significant multivariate effect over time (Wilks’ Lambda = 0.316, F(20,57) = 6.176, ɳ² = 0.684, P < 0.001), indicating significant improvements in the outcome measures across sessions. Within‑group effect sizes ranged from small to moderate (d = 0.02–0.53), with comparable improvements observed in both the VR-supported and face-to-face formats. Qualitative findings further enriched the interpretation of these patterns, yielding two overarching themes: “user-friendly program” and “efficient psychotherapy program.”

**Conclusion:**

Both VR-based and face-to-face positive psychotherapy were associated with improvements in emotional experiences, life satisfaction, and suicidal ideation in individuals with major depressive disorder. VR-based delivery appeared feasible and acceptable, with outcomes comparable to the traditional face-to-face format in this preliminary trial. Further studies with larger samples and longer follow-up are needed to confirm these findings and clarify their clinical implications.

**Trial registration:** This trial was prospectively registered in the Iranian Registry of Clinical Trials (IRCT20201001048893N7, registration date: 16 November 2022, URL: https://irct.behdasht.gov.ir/trial/66424) prior to the enrollment of the first participant.

## Introduction

Major depressive disorder (MDD) represents a prevalent mental health condition [1], distinguished by a consistently low or despondent mood, anhedonia or diminished interest in enjoyable pursuits, sensations of guilt or worthlessness, diminished energy levels, impaired concentration, alterations in appetite, psychomotor retardation or agitation, disruptions in sleep patterns, and contemplation of suicide [2]. According to the latest reports, about 280 million people in the world are suffering from this disease [3]. According to a review study, the prevalence of depression in Iran is also reported to be 15–29% [4].

In 2020, MDD was a significant factor in the global disease burden, contributing to 49.4 million disability-adjusted life-years (DALYs) across the world [5]. One of the main symptoms of depression is disturbance in emotional experiences [6]. Emotional experiences are mental states of experiencing emotions that are related to psycho-neurological changes [7]. Patients with depression experience many negative emotions, such as anger, anxiety, a depressed mood, and hopelessness [8]. Furthermore, emotional blunting emerges as a prevalent symptom among individuals grappling with depression [7,9]. Misplaced emotional experiences in patients with depression can affect their daily lives and relationships [7] and lead to a decrease in life satisfaction and suicide ideation [10,11].

Life satisfaction is a person’s view of the world, which is related to the person’s needs. In fact, it is a cognitive evaluation of life based on the level of achievement toward goals [12]. Depressed people usually do not get any pleasure from their lives, feel worthless, and think themselves helpless; as a result, they have low life satisfaction [13]. In a study using diagnostic data, 63% of individuals with current MDD reported severely impaired satisfaction scores [13]. This low life satisfaction is associated with an increased likelihood of generating and developing suicidal ideation [14].

Suicidal ideation is estimated to be present in 18–58% of individuals with MDD and serves as a major predictor for suicide attempts and deaths [15,16]. This phenomenon poses a substantial economic burden [17]. Jaffe et al. [18] observed in Europe that individuals with suicidal ideation experience higher levels of absenteeism, presenteeism, reduced work productivity, and overall impairment in daily activities compared to those without such thoughts [19].

Multiple conceptual frameworks have been proposed to explain abnormal emotional reactivity in depression. Among the most prominent are the negative potentiation model, which posits heightened reactivity to negative stimuli; the positive attenuation model, which suggests reduced responsiveness to positive stimuli; and the emotion context insensitivity (ECI) model, which proposes a generalized blunting of emotional responses across both positive and negative contexts [20]. Together, these models provide an important conceptual basis for understanding emotional dysfunction in depression.

Disruptions in these emotional processes—particularly the diminished responsiveness to positive stimuli emphasized by the positive attenuation model—may undermine individuals’ ability to experience pleasure, sustain motivation, and evaluate their lives positively [20]. Consequently, individuals with MDD often report lower levels of life satisfaction and psychological well-being and may be more vulnerable to feelings of hopelessness and suicidal ideation [21]. These patterns highlight the importance of interventions that directly target and enhance positive emotional processes and adaptive psychological resources. Positive Psychotherapy (PPT), originally introduced by Seligman and colleagues in the late 1990s, was developed to cultivate positive emotions, personal strengths, and meaning in life, thereby shifting individuals’ focus from deficits toward psychological resources [22]. In this regard, PPT primarily focuses on enhancing positive emotions, strengths, and adaptive cognitive patterns, thereby targeting emotional and cognitive symptoms associated with depression rather than directly addressing its underlying causal mechanisms [22]. Consistent with this symptom‑focused perspective, the present study evaluates changes in psychological well-being and depressive symptoms rather than attempting to test underlying causal mechanisms. In recent years, a growing body of research has identified PPT as a promising therapeutic approach that can enhance psychological well-being and alleviate depressive symptoms in individuals experiencing depression [23,24].

Growing evidence supports the efficacy of positive psychotherapy and related positive psychology interventions (PPIs) in improving mental health outcomes [25–27]. Meta‑analytic evidence indicates that PPIs produce small‑to‑moderate improvements in subjective well‑being and life satisfaction, along with significant reductions in depressive symptoms across both clinical and non‑clinical populations [23,25,26]. However, comparative reviews suggest that the efficacy of PPIs is generally comparable to that of other active psychological treatments rather than consistently superior to them. In particular, systematic reviews indicate that PPIs may represent a viable alternative to established approaches such as cognitive behavioral therapy (CBT), producing broadly similar outcomes for depression and well‑being [28]. Although the findings of this review should be interpreted cautiously due to the limited number of included studies, variability in trial methodologies, and moderate to high risk of bias, the integration of technology in delivering PPIs may offer new opportunities to enhance their efficacy. This highlights the importance of continued research in this area.

Virtual reality (VR) is increasingly used to deliver psychological programs by providing immersive and controllable environments that support psychological processes [29]. Evidence indicates that VR-based interventions can be efficacious for anxiety [29–32] and depressive symptoms [29,30,32–34]. A scoping review by Baghaei et al. (2021) highlighted the growing use of VR for treating depression and anxiety and emphasized its potential to enhance engagement and accessibility [32]. Broader reviews similarly suggest that VR facilitates mechanisms such as emotional processing, behavioral activation, and exposure [30].

VR-based psychological interventions have increasingly been evaluated in comparison with conventional psychotherapy. Meta-analytic and systematic review evidence suggests that VR-based treatments can achieve levels of efficacy comparable to established face-to-face interventions, particularly when VR is used as a delivery format for evidence-based therapeutic approaches [32,33,35,36]. These findings indicate that clinical outcomes are generally comparable rather than clearly superior to traditional face-to-face treatments, suggesting that therapeutic benefits are primarily attributable to the underlying psychological intervention rather than the VR format itself [36,37]. Accordingly, VR is increasingly conceptualized as a technological platform that can enhance engagement, experiential learning, and accessibility, rather than as a standalone therapeutic approach [32,37,38].

Most VR-based interventions for depression have been developed within cognitive-behavioral therapy (CBT) frameworks [29,39]. CBT is a structured and widely implemented psychotherapeutic approach that focuses on identifying and modifying maladaptive cognitions and behaviors through techniques such as cognitive restructuring and behavioral activation [40]. Although CBT is supported by robust meta‑analytic evidence for reducing depressive symptoms [41], it primarily targets maladaptive cognitions and negative symptoms, with limited focus on fostering positive emotions, meaning, and psychological well‑being [42]. Consistently, a recent review found that 35% of VR‑based mental health interventions showed minimal or no improvement in positive emotional or cognitive outcomes [43]. This gap underscores the need for approaches such as positive psychotherapy, which cultivates strengths, positive emotions, and meaning [44,45], yet remains underexplored within VR-based interventions designed to promote psychological well-being in individuals with MDD.

Positive psychotherapy (PPT), which focuses on cultivating positive emotions and strengthening personal capacities [46], has been proposed as an approach for addressing the pervasive negativity and reduced psychological well‑being commonly observed in individuals with MDD [22]. However, the effectiveness of PPT‑based exercises may depend on providing an experientially engaging and emotionally supportive context that facilitates positive emotional processing. VR has increasingly been used to deliver psychological programs by providing immersive and controllable environments that can enhance engagement and experiential involvement [29–32]. Nevertheless, most VR‑based interventions for depression have been developed within cognitive‑behavioral therapy (CBT) frameworks rather than positive‑psychology approaches [29,39]. This gap highlights the need to examine whether VR can effectively serve as a delivery format for PPT, particularly for outcomes related to psychological well‑being such as emotional experiences, life satisfaction, and suicidal ideation. Accordingly, the present study aimed to determine whether delivering PPT through a VR platform produces different effects compared with traditional face-to-face PPT among adults diagnosed with MDD, with a primary focus on well-being-related outcomes, including emotional experiences, life satisfaction, and suicidal ideation. We hypothesized that VR‑based PPT would yield greater improvements in emotional experiences, life satisfaction, and suicidal ideation due to the enhanced experiential immersion and emotional engagement afforded by the VR platform.

### Theoretical framework

Within the context of depression-related emotional dysfunction described earlier, Seligman’s well‑being framework provides a useful perspective for conceptualizing improvements in positive emotional and cognitive functioning. In Seligman’s theoretical framework (2011), well-being is characterized by the amalgamation of cognitive happiness (satisfaction), hedonic happiness (feeling), and eudaimonia (meaning). Well-being is proposed to depend on five key elements denoted as positive emotion, engagement, relationships, meaning, and achievement, succinctly represented by the acronym PERMA. The integration of these PERMA elements is posited to stimulate flourishing, signifying the optimal functioning of individuals, groups, communities, nations, and society as a whole. Augmenting the PERMA elements is proposed as a strategy to enhance overall well-being [47].

Building on this theoretical framework, this study examines the impact of VR-based positive psychotherapy on a triad of well-being indicators: life satisfaction (cognitive well-being), affect (hedonic well-being), and suicidal ideation (a critical marker of distress). These outcomes were selected not as isolated or competing endpoints, but as theoretically coherent components of psychological well-being that map onto distinct facets of the PERMA model [48]. Consistent with the aims of positive psychotherapy, the study focuses on multidimensional indicators of well‑being rather than a single symptom‑based clinical outcome. This approach provides an integrated and conceptually grounded assessment of well‑being, addressing concerns about multiplicity by focusing on complementary dimensions of a unified construct rather than unrelated clinical outcomes.

## Materials and methods

This research adopted a sequential explanatory mixed-methods approach, incorporating both quantitative and qualitative phases, with a slight focus on the quantitative data. This approach facilitated a comprehensive exploration and interpretation of the study topic, enriching the understanding of quantitative findings. The integration of results from both phases enhanced the study’s robustness, providing a nuanced and holistic perspective on the research topic [49].

In this study, following the completion of the quantitative phase, key participants from the intervention group engaged in the qualitative phase. Subsequently, the findings from both phases were integrated during the discussion to provide a deeper understanding of how VR‑based positive psychotherapy influences selected well‑being–related outcomes—namely emotional experiences, life satisfaction, and suicidal ideation—among individuals diagnosed with MDD.

### Quantitative phase

**Fig 1:**
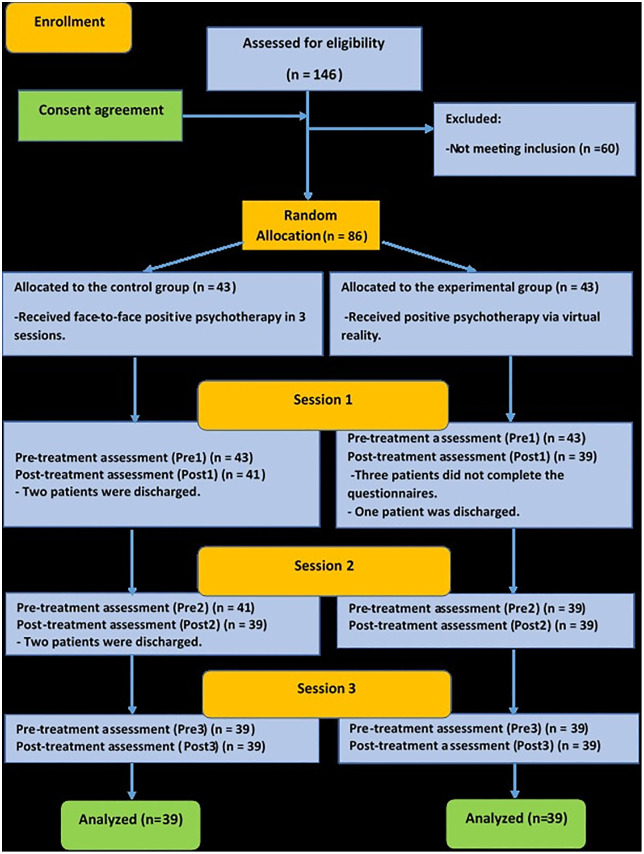
CONSORT flow diagram.

The quantitative phase of this research utilized a randomized controlled trial with a parallel-group design and a 1:1 allocation ratio. The procedures and documentation of all methodologies employed in the trial adhered to the CONSORT guidelines. A completed CONSORT checklist is provided as Supporting Information. Participant flow throughout the trial is presented in the CONSORT flow diagram (Fig 1).

### Participants and settings

The participants in this study were adult patients referred to the psychiatric departments of two general hospitals situated in the southern region of Iran. All participants were diagnosed with unipolar MDD according to the criteria specified in the Fifth Edition of the Diagnostic and Statistical Manual of Mental Disorders (DSM-V) [50]. The diagnosis was additionally verified by a psychiatrist using the Structured Clinical Interview for DSM (SCID-5).

All participants continued routine pharmacological treatment during the study. All patients were prescribed sertraline, a selective serotonin reuptake inhibitor (SSRI) commonly used as a first-line treatment for MDD, within the usual therapeutic range (25–200 mg/day), as determined by the treating psychiatrist. Medication management was part of standard clinical care and was not manipulated by the research team. Participants were not required to initiate, discontinue, or modify their pharmacological treatment in order to participate in the study. Dosages remained stable during the brief intervention period, and the treating psychiatrist was not involved in delivering the psychological interventions or outcome assessments. No systematic differences in medication management were observed between groups; therefore, pharmacotherapy was not considered an experimental component of the study.

To meet the eligibility criteria for participation, participants had to be aged 18 or older, express willingness and preparedness to participate in the study, demonstrate literacy in reading and writing, have a psychiatrist-confirmed diagnosis of major depression, obtain a depression score exceeding 10 on the Patient Health Questionnaire (PHQ-9), report suicidal ideations as documented in the patient’s medical record, and be assessed through the Composite International Diagnostic Interview (CIDI) done by a senior psychiatric nursing student. Additionally, participants were required to have no psychotic or other mental disorders and no debilitating chronic or severe physical illnesses, as indicated in their medical records. This study employed the following exclusion criteria: individuals with psychotic or other mental health disorders other than MDD; debilitating chronic or severe physical illnesses, as indicated in their medical records, that could interfere with psychotherapy; those with a suicide risk requiring immediate hospitalization; individuals experiencing unexpected physical or mental problems during the study; and those unable to actively participate in the study process due to reasons such as slowness of thinking or speech. Additionally, participants expressing reluctance to continue contributing to the study were also excluded. All study procedures were conducted in accordance with the study protocol approved by the institutional ethics committee, which is provided as Supporting Information.

### Randomization and blinding

Patients were randomly allocated to two groups: the intervention group, receiving positive psychotherapy via VR, and the control group, receiving traditional face‑to‑face positive psychotherapy. Randomization was performed using computer-generated allocation sequences. The numbers corresponding to the psychiatric wards were entered into the randomization software, and this software placed the psychiatric wards in either the intervention or control groups completely randomly and by coding. Participants were recruited from the same psychiatric wards across the two hospitals, and post‑randomization checks indicated no systematic imbalance in recruitment sources between the intervention and control groups.

To ensure allocation concealment, the randomization sequence generated for the participating psychiatric wards was kept by an independent researcher who was not involved in patient recruitment or intervention delivery. The allocation codes for the wards were inaccessible to the study team until the wards were assigned to their respective groups. The nurse responsible for participant enrollment was unaware of the ward allocation codes and therefore had no knowledge of which group each ward would be assigned to at the time of enrollment, ensuring concealment.

To minimize bias, data collection, questionnaire interpretation, and data entry and analysis were carried out by external research assistants and statisticians who were blinded to group assignments. In accordance with ethical principles, all patients were informed about the study’s focus on positive psychotherapy; however, participants were not informed about the specific intervention method (VR vs. face‑to‑face) used in the other group.

### Materials and apparatus

A VR headset supplied by SHINECON G06A was used. This VR headset features a 185 x 133 x 100 mm screen and offers a wide field of view from 90° to 100°. It comes with adjustable IPD (interpupillary distance) ranging from 60 to 70 mm, HD resin aspherical lenses with a 40 mm diameter, and a fixed focal length of 37.5–46.5 mm. Additionally, it is compatible with smartphones running iOS, Android, or Windows Phone, accommodating screen sizes from 4.7 to 6 inches. We opted for this headset due to its notable features, including a wireless system, lightweight design (276 grams), comfort, and user-friendly interface. Furthermore, the headset is compatible with various mobile phone models and comes equipped with a controller (B01) to provide an immersive and captivating VR experience. The immersive 360° videos were designed based on the main concepts and fundamentals of positive psychotherapy by a team of experts, including professionals in the field of Unity game development, a C# programming language expert, and a 3D modeling specialist.

### Intervention condition: VR-based positive psychotherapy

This intervention condition involved receiving three 30-minute VR sessions every other day. The sessions were designed based on Seligman’s positive psychotherapy [51], which is a suitable approach for enhancing well-being, fostering positive emotions, and promoting personal strengths in individuals. The number of sessions was adapted from the original protocol to enhance feasibility, consistent with previous evidence indicating that brief positive psychotherapy interventions can produce meaningful improvements in psychological well-being [25,52]. The sessions’ content was developed by a team of psychologists and psychiatric nurses under the supervision of the first author. They included 3D images, videos, sounds, and one or two exercises related to the contents of each session, which were prepared based on core positive psychotherapy concepts and practices, including kindness, gratitude, appreciation, identification of strengths, and goals. The intervention components were selected based on core mechanisms emphasized in positive psychotherapy, prioritizing elements that are theoretically central and well suited for experiential implementation within immersive VR environments. The content of the sessions is presented in Table 1.

**Table 1:** Topics of sessions.

Contents	Session
• Instructing how to use the 3-dimensional glass, headset, and related accessories. • Expressing the goals and plans of the program. • Expressing empathy with the patient and a supportive presence for the patient. • Expressing love and value toward patients. • Asking the patient to describe her or his problem and negative feelings. • Encouraging participants to face negative thoughts. • Mentioning positive sentences • Encouraging patients to practice self-love, self-care, and self-acceptance. • Encouraging to schedule enjoyable, meaningful, and purposeful activities and go through them. • Exercising deep breathing and muscle relaxation	1
• Expressing supportive presence for the patient. • Expressing love and value toward patients. • Encouraging participants to face negative thoughts • Mentioning positive sentences • Mentioning positive sentences. • Encouraging participants to mention personal strengths, gratitude for them, and life-positive events. • Encouraging patients to keep in mind the loved ones\ support received and exercising gratitude for them. • Focusing on and appreciating nature’s beauties. • Getting to know the meaning of life. • Exercising deep breathing and muscle relaxation.	2
• Expressing supportive presence for the patient. • Expressing love and value toward patients. • Encouraging participants to face negative thoughts. • Mentioning positive sentences • Encouraging participants to forgive transgressors • Encouraging participants to act with kindness related to themselves and others or to do good deeds for others • Encouraging participants to have hope and optimism for the future • Exercising deep breathing and muscle relaxation.	3

**Fig 2:**
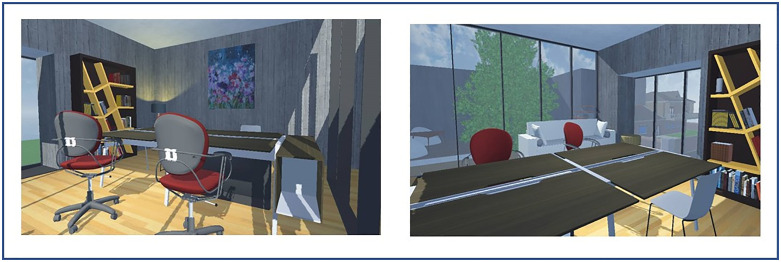
Screenshots of the consulting room.

**Fig 3:**
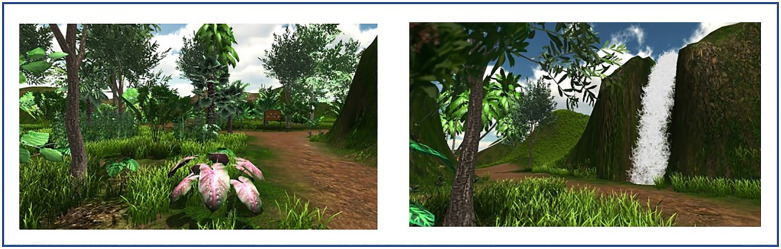
Screenshots of the natural setting.

Participants in the VR-based positive psychotherapy group wore a VR headset and initiated the session using a handheld controller. The VR program provided patients with immersive experiences in two distinct environments: a simulated consulting room designed to create a supportive and reflective atmosphere (see Fig 2), followed by a natural environment (e.g., forest, mountains, sky, and waterfall scenes), offering a tranquil and calming experience (see Fig 3).

Prior to the VR sessions, a trained psychiatric nurse (the second author) provided participants with brief explanations about the nature of the study, the intervention procedure, and the use of the VR headset and related equipment. Participants were instructed to focus on the session content and actively participate in the scheduled activities of the intervention. They were also encouraged to ask any questions to ensure familiarity with the equipment before the session began.

After the initial setup of the VR equipment, participants proceeded with the session independently. The psychiatric nurse remained in the room solely to supervise the procedure, ensure participant safety, and provide technical assistance if needed. Importantly, the nurse did not provide any psychological guidance or intervention during the sessions.

The VR intervention was designed as a guided, non‑interactive experience. The virtual environment did not include an avatar or any visual representation of the therapist. All intervention content and instructions were delivered exclusively through pre‑recorded audio narration embedded within the VR program, which guided participants through the activities and prompted reflection on personal experiences, such as recalling meaningful life events, identifying personal strengths, and expressing gratitude.

Participants could explore the virtual environment through natural head movements, allowing them to observe the surrounding immersive scene from different directions. A handheld controller was used exclusively to initiate the intervention and to transition between predefined audio‑visual parts within the virtual scenes.

Participants’ verbal responses were not processed by the VR program, and the virtual environment did not dynamically respond to user input. Instead, participants engaged in introspective reflection while following the audio instructions within the immersive visual scenes.

### Control condition: face-to-face positive psychotherapy

The participants in the control group received three 30-minute face-to-face positive psychotherapy sessions every other day. The psychiatric nurse (the second author) provided these patients with consultations and exercises based on positive psychotherapy techniques, similar to what was provided for the intervention group. The in-person sessions were held in a private and quiet room in the psychiatric ward of the hospital.

### Recruitment and procedure

Participant recruitment was conducted between January 20 and September 22, 2023. Outcome data were collected immediately before the first session (baseline) and after each of the three intervention sessions. No additional follow-up assessments were conducted after the final session.

At the outset, the research team obtained demographic and clinical data for 146 patients who had been diagnosed with unipolar MDD by a psychiatrist, based on DSM-V criteria. Following this, a psychiatric nurse (the third author) explained the aims of the study to the patients and inquired whether they were willing to participate in the screening process. She conducted screenings for the individuals who expressed interest until the target number of 86 participants was reached. All participants were informed that their participation was entirely voluntary, and they provided written informed consent.

### Measures

In this study, sociodemographic and clinical characteristics were assessed using standardized forms developed by the research team. Data were collected on participants’ age, gender, marital status, educational level, and employment status, along with the duration of depressive symptoms and any family history of psychiatric disorders. The study utilized the following outcome measures:

### Primary outcome

The primary outcome assessed in this study was emotional experience, given its significant influence on life satisfaction and suicidal ideation [10,11]. In line with the PERMA framework, emotional experience was prespecified as the primary outcome, whereas life satisfaction and suicidal ideation were considered secondary outcomes. Prespecifying a primary outcome helps clarify interpretation when multiple endpoints are examined [53].

To evaluate emotional experiences, the Positive and Negative Affect Schedule (PANAS) was employed. Originally developed by Watson et al. (1988), PANAS consists of 20 self-report items, equally distributed between positive and negative affect subscales. Participants rated their emotional states on a 5-point Likert scale ranging from ‘Very slightly or not at all’ to ‘Extremely,’ with scores for each subscale ranging from 10 to 50. Previous research by Watson and Clark (1988) demonstrated strong internal consistency for PANAS, with Cronbach’s alpha coefficients ranging from 0.86 to 0.90 for positive affect and 0.84 to 0.87 for negative affect across different time frames. The instrument also showed satisfactory test-retest reliability over an 8-week period, with correlation coefficients varying between 0.47 and 0.68 for positive affect and 0.39 to 0.71 for negative affect. Convergent validity was supported through its correlation with the Beck Depression Inventory, yielding coefficients between 0.36 and 0.58 over the ‘past few weeks’ [54]. In the Iranian context, Zargar et al. reported a Cronbach’s alpha of 0.80 for the negative affect subscale, affirming its reliability [55]. In the present study, PANAS exhibited acceptable internal consistency, with a Cronbach’s alpha of 0.78 for both the positive and negative affect subscales.

### Secondary outcome

In this study, participants’ life satisfaction was assessed using the Satisfaction With Life Scale (SWLS), developed by Diener et al. (1985). This instrument consists of five items rated on a seven-point Likert scale, ranging from 1 (‘completely disagree’) to 7 (‘completely agree’), yielding a total score between 5 and 35. Score interpretations classify individuals into categories such as very high (30–35), high (25–29), average (20–24), slightly below average (15–19), dissatisfied (10–14), and strongly dissatisfied with life (5–9). Diener et al. (1985) evaluated the psychometric properties of SWLS among 176 undergraduate students, reporting a test-retest reliability of 0.82 over two months and a Cronbach’s alpha of 0.87. The scale demonstrated acceptable validity, with correlations of 0.50 and 0.37 with positive and negative affect scales, respectively [56]. In an Iranian context, Maroufizadeh et al. (2016) confirmed the reliability and validity of SWLS, with confirmatory factor analysis supporting a single-factor structure. Additionally, significant negative correlations were found between SWLS and the Hospital Anxiety and Depression Scale (HADS) for anxiety (r = −0.410) and depression (r = −0.434), indicating satisfactory convergent validity. The study also reported a Cronbach’s alpha of 0.887 for the scale [57]. In the current study, the internal consistency of SWLS, as measured by Cronbach’s alpha, was 0.91, demonstrating strong reliability.

In this study, the Brief Suicide Cognitions Scale (B-SCS) assessed patients’ suicidal ideation using a 6-item self-report scale. The B-SCS measures the suicidal belief system, encompassing enduring hopelessness related to self-perceived unlovability, emotional unbearability, and life problems perceived as unsolvable. The Likert-scaled items (1–5) focus on unlovability, unbearability, and unsolvability, yielding scores between 6 and 30. The B-SCS demonstrated strong reliability, validity, unidimensional factor structure, and predictive value in clinical settings [58]. The B-SCS showed robust internal consistency reliability (Cronbach’s alpha: 0.84–0.91) and strong test-retest reliability (0.84) in Rudd and Bryan’s study. In Iran, Batouei et al. validated the scale, revealing two significant subscales: unlovability and intolerability. Convergent validity was supported by correlations with Beck’s Suicide Ideation (0.57), while concurrent and divergent validity were indicated by correlations with Beck’s Hopelessness (0.62) and Beck’s Depression Inventory (−0.62), respectively [59]. The overall scale exhibited good internal consistency, with a reported Cronbach’s alpha of 0.85 in previous studies. In the present study, the reliability assessment indicated a Cronbach’s alpha of 0.82, confirming the acceptable internal consistency of the scale.

### Feasibility, adherence, satisfaction, and sickness with VR-based positive psychotherapy

Feasibility in this study referred to the practicality of implementing the VR‑based positive psychotherapy intervention and conducting the study procedures within the clinical setting. Feasibility was assessed based on the proportion of eligible participants who enrolled in the study and completed it. A minimum of 70% adherence to the study protocol was considered indicative of acceptable feasibility [60]. Adherence to the intervention was determined by the number of modules and exercises completed by participants, as reported by themselves. Full adherence was defined as completion of all presented modules across the three sessions, along with providing feedback on the corresponding exercises.

In this study, two questionnaires, the Credibility/Expectancy Questionnaire (13) (Borkovec and Nau, 1972) [61,62], and the Virtual Reality Sickness Questionnaire (VRSQ) (Kim et al., 2018) [63], were used to assess satisfaction with the intervention and motion sickness in VR environments, respectively. The CEQ is a self-report measure comprising 5 items, with ratings on a Likert scale from 1 (indicating low/poor treatment expectation) to 10 (indicating high/favorable treatment expectation) [62]. In line with the present study, we employed a modified version of the CEQ [64] with five dimensions, including logical coherence (“How logical does this psychological program seem to you?”), overall satisfaction (“How satisfied are you with the psychological program?”), likelihood of recommending the program (“How confident would you be in recommending this psychological program to a friend who is experiencing a similar situation?”), perceived utility (“How useful do you think this program has been for you?”), and discomfort experienced (“How annoying or uncomfortable has this program been for you?”). Responses were assessed on a scale ranging from 0 (“not at all”) to 10 (“completely”). Both study groups completed this assessment at the conclusion of the third session. The VRSQ comprises nine items rated on a four-point Likert scale (0 = not at all, 3 = severe) and is divided into two subscales: the oculomotor system and disorientation. It assesses various symptoms such as fatigue, general discomfort, eye fatigue, difficulty concentrating, headache, motion sickness, blurred vision, feeling lightheaded, dizziness, and vertigo experienced during VR exposure [63]. This scale was completed by the patients in the intervention group after the third session. Both of these questionnaires have demonstrated good psychometric properties in previous research [61–63]. In this study, the VRSQ and the adapted CEQ demonstrated Cronbach’s alpha coefficients of 0.78 and 0.71, respectively.

### Sample size

The sample size was calculated a priori based on the randomized controlled trial by Pietrowsky and Mikutta [65]. In that study, post‑treatment positive affect scores (PANAS) differed significantly between groups, with means of 26.89 (SD = 6.35) and 19.75 (SD = 5.92). The between‑group mean difference of 7.14, derived from these post‑treatment values, and the corresponding standard deviations were used as inputs for the present power analysis. Positive affect was therefore selected as the principal endpoint among our three outcomes (affect, life satisfaction, and suicidal ideation). Using MedCalc with α = 0.01 and power = 0.99, the required sample size was 37 participants per group; assuming 15% attrition, the target sample size was set at 43 per group (total N = 86).

### Statistical analysis

Statistical analyses were conducted using IBM SPSS Statistics (Version 22.0; IBM Corp., Armonk, NY, USA). Descriptive statistics (means, standard deviations, and frequencies) were calculated to summarize demographic and clinical characteristics and to evaluate feasibility, adherence, participant satisfaction, and any adverse effects associated with the VR-based positive psychotherapy intervention. Baseline equivalence between the VR-based positive psychotherapy (VR-PPT) group and the face-to-face positive psychotherapy group was assessed using independent samples t-tests for continuous variables and chi-square (χ²) tests for categorical variables. All analyses were performed on a complete-case basis; participants with missing outcome data at the relevant time points were excluded from the corresponding analyses.

In this study, the independent variable was the mode of positive psychotherapy, categorized as VR-based positive psychotherapy (VR-PPT) or face-to-face positive psychotherapy. The dependent variables included positive affect (PANAS-PA), negative affect (PANAS-NA), life satisfaction (SWLS), and suicidal ideation (B-SCS), representing emotional experiences, life satisfaction, and suicidal ideation. These outcomes were measured at baseline and immediately before and after each intervention session.

Data normality was evaluated using the Kolmogorov–Smirnov and Shapiro–Wilk tests, and homogeneity of variances was examined using Levene’s test. The Kolmogorov–Smirnov test indicated no significant departures from normality for any variable. Although the Shapiro–Wilk test showed a mild deviation from normality for the life satisfaction variable, inspection of skewness and kurtosis values indicated that these departures were minimal. Considering the equal group sizes (n = 39) and strong empirical evidence that parametric procedures are robust against small violations of normality—particularly when such deviations are limited to slight skewness or kurtosis [66,67]— parametric analyses were considered appropriate for subsequent statistical procedures. Detailed normality results are provided in S1 File.

To examine intervention effects while accounting for multiple dependent variables, a 2 (Group: VR-PPT vs. face-to-face PPT) × 6 (Time: pre-and post-sessions 1, 2, and 3) mixed-model repeated-measures multivariate analysis of variance (MANOVA) was conducted. In this model, time was treated as the within-subject factor and group as the between-subject factor. Following significant multivariate effects, univariate repeated-measures ANOVAs were performed for each outcome variable to identify specific sources of change. The assumption of sphericity was evaluated using Mauchly’s test, and when violated, Greenhouse–Geisser corrections were applied. Post hoc comparisons were conducted to explore significant within-group changes across measurement points.

Effect sizes were reported to provide a more comprehensive evaluation of the intervention effects beyond statistical significance testing. Partial eta-squared (η²p) was used to estimate effect sizes for ANOVA analyses, and Cohen’s d was calculated for pairwise comparisons. Cohen’s conventional benchmarks (0.2 = small, 0.5 = medium, and 0.8 = large) were used to aid interpretation of effect magnitudes [68]. The magnitude of these effect sizes was considered when interpreting the findings. Statistical significance was set at p < 0.05.

### Qualitative phase

Participants for the qualitative phase were selected using purposive sampling to ensure the inclusion of individuals with rich and direct experience of the VR-based positive psychotherapy program. In line with Patton’s (2014) recommendations, purposive sampling was employed to intentionally select “information-rich cases” capable of providing in-depth insight into the phenomenon under study rather than achieving statistical representativeness [69]. Accordingly, participants were eligible if they had: (1) completed the full VR intervention, and (2) expressed willingness to engage in an in depth interview and articulate their lived experiences. The second researcher (FAH) established telephonic communication with the participants, delivering comprehensive explanations regarding the purpose and scope of this study segment. Subsequently, for individuals expressing interest in study participation, she requested the completion and return of the provided informed consent through WhatsApp.

Each interview had a duration ranging from 30 to 45 minutes, and data were collected through semi-structured interviews. At the commencement of each interview, the researcher (the second author) posed some general and open-ended questions: “What is your opinion regarding the sessions and exercises delivered to you through the VR headset?” and “What changes did you perceive within yourself following these sessions?” Subsequently, the interview progressed to more detailed questions, such as “Why were you influenced by these trainings and exercises?.” If necessary, additional questions were directed towards the participant to elicit further clarification on her response and the subject mentioned. When applicable, probing questions like “Could you elaborate further?” “May you provide an example for better comprehension?” and “What does it mean?” were used.

Transcripts were reviewed several times immediately after each interview and then transcribed. Data analysis was conducted using the conventional content analysis approach proposed by Graneheim and Lundman (2009) [70]. This process involved several key stages, including identifying the unit of analysis, systematically coding the data, categorizing based on patterns of similarity and difference, condensing the data, and ultimately extracting overarching themes. Data collection continued until thematic saturation was reached, consistent with established qualitative standards [71]. Saturation occurred after 13 interviews, at which point no new codes, categories, or thematic variations were emerging from the data. Therefore, no further participants were enrolled in the qualitative phase. This sampling strategy and sample size are appropriate for exploratory qualitative studies embedded within mixed-methods designs.

To ensure the trustworthiness of the findings, Guba and Lincoln’s evaluative criteria were applied [72]. Credibility and confirmability were reinforced through prolonged engagement with participants over approximately seven months. To enhance credibility, initial coding underwent independent review by external research team members and three qualitative experts unaffiliated with the study. Additionally, member checking was conducted, wherein participants assessed the coded transcripts to ensure alignment with their lived experiences. Transferability was addressed by providing a detailed account of the research methods and findings. After completing the qualitative data analysis, the results of this phase were assessed and compared with those of the quantitative phase to determine homogeneity or heterogeneity.

### Ethical considerations

The present study was conducted in accordance with the ethical guidelines outlined in the Declaration of Helsinki. Ethical approval was granted by the Ethics Committee of Shiraz (IR.SUMS.NUMIMG.REC.1401.059), and the study was officially registered in the Iranian Registry of Clinical Trials (IRCT20201001048893N7, registration date: 16 November 2022, https://irct.behdasht.gov.ir/trial/66424). Ethical considerations, including maintaining participant confidentiality and obtaining written informed consent, were rigorously upheld. Participants were fully informed about the study objectives, procedures, and their right to withdraw at any stage without consequence. Regarding data availability, relevant datasets can be accessed upon reasonable request by contacting the corresponding author via email.

## Results

### Quantitative phase

The quantitative analyses examined changes in well‑being–related outcomes, including positive affect, negative affect, life satisfaction, and suicidal ideation, across the study period. Among the 86 patients deemed eligible for the study, 78 participants successfully completed the trial, with an equal distribution between the intervention (n = 39) and control (n = 39) groups. The primary analyses are based on participants with available outcome data at each time point (complete-case numbers). A total of 8 patients were excluded, comprising four individuals from each group. In the first post-treatment assessment of the intervention group, three patients didn’t complete the questionnaires, and one of them was discharged. Moreover, four patients were excluded from the control group in the first (two patients) and second (two patients) post-treatment assessments because of discharging [Refer to the CONSORT diagram (Fig 1) for a detailed overview of the participant flow].

The mean age of the participating patients was 35.38 ± 8.23 years. The participants had an average duration of depressive symptoms of 62.31 ± 54.29 months. The majority were female (91.03%), married (57.69%), and held an undergraduate degree (52.70%) (Table 2). Additionally, half of the participants (50%) reported a family history of psychiatric disorders. No significant differences were observed between the intervention and control groups in terms of demographic and clinical characteristics (Table 2).

**Table 2:** Sample characteristics (n = 78).

Characteristic	Total N = 78	Control N = 39	Experimental N = 39	Value	P-Value
**Age, M (SD)**	35.38 (8.23)	37.18 (7.46)	33.59 (8.67)	−1.96 (t)	0.054
**Duration of depressive symptoms, M (SD)**	62.31 (54.29)	58.26 (60.81)	66.36 (47.35)	0.66 (t)	0.51
**Gender, n (%)**					
Male	7 (8.97%)	3 (7.69%)	4 (10.26%)	0.16 (F)	0.69
Female	71 (91.03%)	36 (92.31%)	35 (89.74%)		
**Marital status, n (%)**					
Single	25 (32.05%)	10 (25.64%)	15 (38.46%)	1.56 (F)	0.46
Married	45 (57.69%)	25 (64.10%)	20 (51.28%)		
Divorced/ Widowed	8 (10.26%)	4 (10.26%)	4 (10.26%)		
**Educational level, n (%)**					
From primary education to diploma	21 (28.38%)	11 (29.73%)	10 (27.03%)	3.87 (F)	0.14
Undergraduate degree	39 (52.70%)	16 (43.24%)	23 (62.16%)		
Postgraduate degree	14 (18.92%)	10 (27.03%)	4 (10.81%)		
**Occupation, n (%)**					
Unemployed	22 (28.20%)	8 (20.51%)	14 (35.90%)	10.04 (F)	0.07
Housekeeper	33 (42.31%)	16 (41.03%)	17 (43.59%)		
Employed	14 (17.95%)	10 (25.64%)	4 (10.26%)		
Retired	1 (1.28%)	1 (2.56%)	0 (0%)		
College student	5 (6.41%)	4 (10.26%)	1 (2.56%)		
Others	3 (3.85%)	0 (0%)	3 (7.69%)		
**Family history of psychiatric disorders, n (%)**				1.28 (χ2)	0.26
Yes	39 (50%)	17 (43.59%)	22 (56.41%)		
No	39 (50%)	22 (56.41%)	17 (43.59%)		

Note: M (SD): mean (standard deviation); n (%): number (percent).

The repeated measures MANOVA analysis demonstrated a statistically significant main effect of time (Wilks’ Lambda = 0.316, F(20,57) = 6.176, ɳ^2^ = 0.684, p < 0.001), suggesting that the outcome variables underwent considerable changes over the study period. However, the main effect of the group did not reach statistical significance (Wilks’ Lambda = 0.918, F(4,73) = 1.633, ɳ^2^ = 0.082, p = 0.175), implying that the overall differences between the intervention and control groups were not significant. Additionally, the interaction effect between time and group was not significant (Wilks’ Lambda = 0.658, F(20,57) = 1.483, ɳ^2^ = 0.342, p = 0.124), indicating that the pattern of change over time was similar across both groups.

The results of the univariate analyses revealed a significant main effect of group on suicidal ideation (F[1,76] = 5.158, ɳ^2^ = 0.064, p = 0.026), indicating a meaningful difference between the groups in terms of the marginal mean of suicidal ideation. However, no significant group effects were observed for positive affect (F[1,76] = 0.613, ɳ^2^ = 0.008, p = 0.436), negative affect (F[1,76] = 0.208, ɳ^2^ = 0.003, p = 0.650), or life satisfaction (F[1,76] = 0.636, ɳ^2^ = 0.008, p = 0.428). Additionally, significant main effects of time were detected for positive affect (F[5,268.001] = 5.366, ɳ^2^ = 0.066, p = 0.001), negative affect (F[5,258.389] = 22.153, ɳ^2^ = 0.226, p < 0.001), life satisfaction (F[5,242.488] = 24.800, ɳ^2^ = 0.246, p < 0.001), and suicidal ideation (F[5,271.372] = 17.947, ɳ^2^ = 0.191, p < 0.001). These findings indicate that, independent of group differences, there were significant changes over time in positive and negative affect, life satisfaction, and suicidal ideation.

Because the intervention effect in longitudinal designs is reflected in the group × time interaction, this interaction was examined to determine whether the trajectories of change differed between the two groups. The interaction effect between group and time for positive affect, negative affect, life satisfaction, and suicidal ideation was not statistically significant (Table 3). This indicates that the patterns of change in these variables over time were similar across both groups, with no significant differences in their trajectories based on group membership.

**Table 3:** One-way repeated measures MANOVAs, F-ratios, p values, and partial η^2^.

Measures	F (df)	P value	ɳ^2^
**Between subjects**			
**Group**			
**Positive affect (PANAS)**	0.613 (1,76)	0.436	0.008
**Negative affect (PANAS)**	0.208 (1,76)	0.650	0.003
**Satisfaction with life (SWLS)**	0.636 (1,76)	0.428	0.008
**Suicide ideation (B-SCS)**	5.158 (1,76)	0.026	0.064
**Within subjects**			
**Time**			
**Positive affect (PANAS)**	5.366 (5,268.001)	0.001	0.066
**Negative affect (PANAS)**	22.153 (5,258.389)	P < 0.001	0.226
**Satisfaction with life (SWLS)**	24.800 (5,242.488)	P < 0.001	0.246
**Suicide ideation (B-SCS)**	17.947 (5, 271.372)	P < 0.001	0.191
Time*group			
**Positive affect (PANAS)**	0.606 (5,268.001)	0.638	0.008
**Negative affect (PANAS)**	1.663 (5,258.389)	0.169	0.021
**Satisfaction with life (SWLS)**	1.151 (5,242.488)	0.330	0.015
**Suicide ideation (B-SCS)**	1.342 (5, 271.372)	0.258	0.017

Note: df = degrees of freedom; B-SCS = Brief Suicide Cognitions Scale; PANAS = Positive and Negative Affect Schedule; SWLS = Satisfaction with Life Scale. Higher scores on Positive Affect and SWLS indicate more favorable outcomes, whereas higher scores on Negative Affect and B‑SCS indicate less favorable outcomes.

Pairwise comparisons between pre-treatment and post-treatment assessments indicated that both groups experienced improvements in positive affect, negative affect, life satisfaction, and suicidal ideation following the intervention. The means, standard deviations, and Cohen’s d effect sizes for the dependent variables across different measurement points are summarized in Table 4. The magnitude of the within-group effect sizes ranged from small to moderate across assessment points (d = 0.02–0.53), indicating generally modest changes in well-being-related outcomes in both groups over the course of the study.

**Table 4:** Repeated measures MANOVAs, means (SDs), and effect sizes (Cohen’s d).

Measure		First session			Second session			Third session		
		Pretreatment M (SD)	Posttreatment M (SD)	Pre-post effect size, d	Pretreatment M (SD)	Posttreatment M (SD)	Pre-post effect size, d	Pretreatment M (SD)	Posttreatment M (SD)	Pre-post effect size, d
**Positive affect (PANAS)**	**Control**	28.43(8.16)	30.67(8.61)	0.27	29.74(8.79)	31.08(9.32)	0.15	30.97(8.84)	31.13(8.42)	0.02
	**Experimental**	27.49(7.99)	29.23(8.21)	0.21	28.33(8.28)	28.95(8.25)	0.07	28.87(8.93)	30.79(9.52)	0.21
**Negative affect (PANAS)**	**Control**	29.10(7.74)	26.72(7.54)	0.31	24.23(6.89)	23.56(6.85)	0.10	23.82(7.46)	23.38(7.78)	0.06
	**Experimental**	29.10(9.53)	26.36(9.16)	0.29	26.18(9.33)	25.28(9.57)	0.09	25.61(10.17)	23.10(9.55)	0.25
**Satisfaction with life (SWLS)**	**Control**	16.15(8.68)	18.43(8.87)	0.26	18.92(9.62)	20.77(9.94)	0.19	20.13(9.92)	21.33(10.41)	0.12
	**Experimental**	14.77(8.82)	18.02(8.81)	0.37	16.54(8.74)	18.56(8.83)	0.23	17.92(9.72)	20.31(9.73)	0.24
**Suicide ideation (B-SCS)**	**Control**	14.56(5.23)	11.87(4.93)	0.53	11.61(4.87)	10.90(4.87)	0.14	11.46(4.88)	10.64(5.03)	0.16
	**Experimental**	16.56(6.91)	14.67(6.50)	0.28	15.20(6.75)	14.33(6.59)	0.13	13.87(5.77)	12.46(6.50)	0.23

Note: M (SD) = mean (standard deviation); d = Cohen’s d; B-SCS = Brief Suicide Cognitions Scale; PANAS = Positive and Negative Affect Schedule; SWLS = Satisfaction with Life Scale. Higher scores on Positive Affect and SWLS indicate more favorable outcomes, whereas higher scores on Negative Affect and B‑SCS indicate less favorable outcomes.

**Fig 4:**
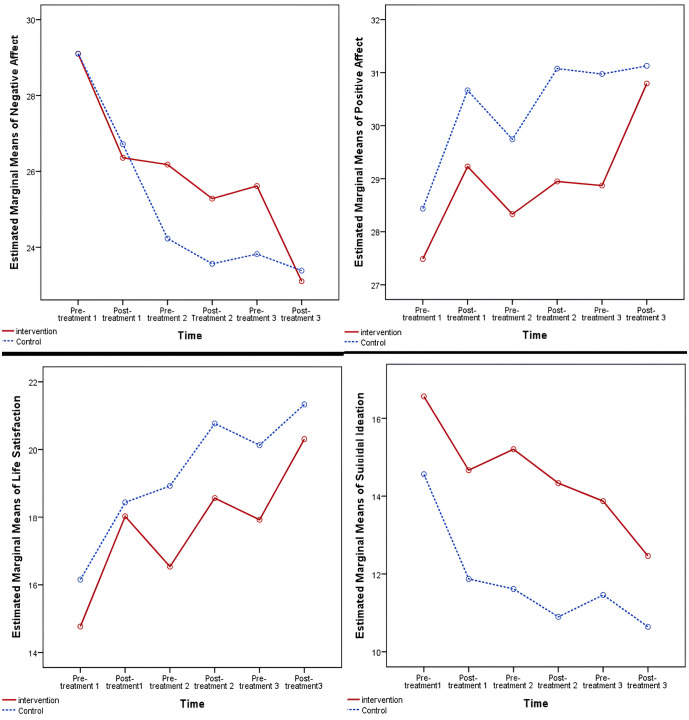
Estimated changes in negative and positive affects, satisfaction of life, and suicidal ideation in the pre-treatment and post-treatment measurements.

The means of positive and negative affect, life satisfaction, and suicidal ideation for both the experimental and control groups are illustrated in Fig 4.

Fig 4 illustrates the trajectories of the study variables across assessment points for the intervention and control groups. Overall, both groups exhibited comparable patterns of change over time. Notably, in the plot depicting negative affect, the plotted trajectories may appear non‑parallel at certain points; however, these visual deviations are inconsistent across sessions. Importantly, the magnitude of the between‑group differences in means at corresponding time points was minimal relative to the within‑group variability, and the within‑session effect sizes were similarly small in both groups. Under such conditions, fluctuations in the plotted means can produce visual deviations in the trajectories without indicating substantively different temporal trends. Accordingly, the pattern observed in Fig 4 is consistent with broadly similar trajectories of change in the two groups, in line with the non‑significant time × group interaction effects.

Individual trajectories and raw data values for all outcome measures are presented in the supplementary Spaghetti plots (S2–S5 Figs), which illustrate the variability in participant-level responses over time within each study group.

Out of the 43 participants who underwent VR-based positive psychotherapy, 39 (90.69%) successfully completed the post-assessments at T3. A total of three patients were excluded from the study for not finishing the questionnaires, and one was removed due to early discharge. Of the 39 patients who completed the post-assessments, 32 (82.05%) fully adhered to the intervention by completing all session modules and providing feedback on the exercises. The remaining participants showed partial adherence, with seven finishing the modules but not submitting feedback. In the control group, all 43 participants began the study, with 39 (90.69%) completing the post-assessments at T3. The other four were excluded due to early discharge.

In both the intervention and control groups, satisfaction levels were notably high, with item scores in the areas of logic, satisfaction, recommendation, and utility being 8 or higher on a 0–10 scale. Additionally, participants did not perceive the intervention as uncomfortable, as their ratings in the Uncomfortableness area were below 1 on the 0–10 scale (refer to Table 5). The t-test results revealed no significant differences between the mean scores of various satisfaction areas between the intervention and control groups (see Table 5).

**Table 5:** T-test statistics, means, and SDs: satisfaction with the intervention scale (adapted version of the Borkovec and Nau inventory).

	Intervention, M (SD)	Control, M (SD)	T-test statistic	P-value
**Logic**	8.13 (2.41)	8.05 (1.97)	0.10	0.92
**Satisfaction**	8.55 (1.97)	8.79 (2.58)	−0.71	0.48
**Recommendation**	8.29 (2.15)	8.42 (2.28)	−0.46	0.65
**Utility**	8 (2.24)	8.16 (2.14)	−0.60	0.55
**Uncomfortableness**	0.71 (1.50)	0.61 (1.82)	0.27	0.78

The VR sickness was assessed in the intervention group after completing the intervention sessions. The average total score of intervention participants on the VRSQ was 7.93 (SD = 11.45), indicating no noticeable or severe VR disease with the program among participants in the intervention group.

### Qualitative phase

To deepen the understanding of the quantitative results, a qualitative phase was added to the study. In this phase, 13 individuals (9 females and 4 males) from the intervention group were interviewed using a semi-structured format via phone. The ages of the participants varied between 18 and 46 years. Table 6 presents the demographic details of the participants involved in this phase.

**Table 6:** Demographic characteristics of the participants.

No	Age	Gender	Education	Job
P1	31	Female	MSc	Homekeeper
P2	30	Female	BSc	Employee
P3	27	Female	BSc	Employee
P4	25	Female	Diploma	Homekeeper
P5	32	Female	Diploma	Homekeeper
P6	34	Female	Diploma	Homekeeper
P7	34	Male	High school	Self-employed
P8	18	Female	Secondary school	Homekeeper
P9	21	Female	BSc student	Student
P10	34	Male	MSc	Employee
P11	32	Female	High school	Homekeeper
P12	46	Male	BSc	Employee
P13	43	Male	Diploma	Self-employed

Two main themes emerged from the qualitative data, including “user-friendly program” and “efficient psychotherapy program.” The main themes, categories, and subcategories derived from the interviews are displayed in Table 7.

**Table 7:** Main themes, categories, and sub-categories in the qualitative stage.

Themes	Categories	Sub-Categories
User-friendly Program	Satisfactory design	High-quality program
		Faultless program
		Desirable virtual environment
	Client-centricity	Empathy with the client
		Client-centric design
		Client privacy preservation
Efficient Psychotherapy Program	Applicable psychotherapy method	Applicability
		Useful adjunctive psychotherapy methods
	Psychological well-being developer	Spirit improvement
		Positivity induction
		Coping improvement
		Negativity reduction
		Self-connection enhancement

- **User-friendly program**

Two categories of this theme were “satisfactory design” and “client-centricity.” The participants believed that the design of the VR program was desirable and satisfactory. They also found the VR program had a constructive focus on them.

- Satisfactory design

The participants in the intervention group clearly mentioned their satisfaction with the VR program. The participants described the VR program as a high-quality and faultless program that motivated them to remain engaged with the VR sessions.

*“In my opinion, the virtual reality program was excellent, and it had no issues or limitations; that’s why I truly used it” (P3).*

*“The software itself was complete in every aspect" (P2).*

A participant pointed out that the technical aspects, including the excellent audio and visual quality, were satisfying, which contributed to her preference for using this program.

*“The sound and image quality were good and satisfying, and in my opinion, this made me enjoy the program, leading to a greater inclination to use it” (P9).*

They also believed that the program provided them with an opportunity to use a desirable, virtual environment, which improved their spirits and helped them feel healthy and positive. They recognized that delivering the psychotherapy program in a desirable nature-based virtual environment has a better effect on the learning of the participants. Two participants stated:

*“Just by seeing the lush nature, water, and waterfalls and walking beside them, I thought that, like healthy individuals, I could enjoy the beautiful nature and have positive feelings. It was excellent. Utilizing this beautiful natural environment was very helpful in uplifting my spirits. The things mentioned in the program, such as expressing gratitude, were also wonderful in this atmosphere and had a positive impact on me” (P7).*

- Client-centricity

The participants perceived a kind of empathy from the virtual psychotherapist while using the program. They felt that they were understood and that their suffering was validated.

*“In the sessions we had, we felt a sense of empathy. For instance, when he said, “I understand; it is just like that; you’re right...” you felt that you were empathized with and that your sadness and suffering were validated, and for that reason, I think it had an effect on me and was a positive step in my treatment process" (P4).*

Moreover, they believed that the program had a client-centric design, as the client’s main concerns and challenges were considered and addressed in the program.

*“The teachings focused on important aspects that were actually my important challenges” (P5).*

*“In my opinion, this program was truly a very good option since it was designed for individuals with depression and to meet their needs” (P6).*

The participants found that their privacy was preserved while using this program.

*“In my view, this program makes the person more comfortable because someone may not feel comfortable talking face-to-face with a counselor about his issues or having others become aware of his problems" (P8).*

- **Efficient psychotherapy program**

The participants in the intervention group clearly experienced the VR interventions as efficient. Categories associated with this main theme were “applicable psychotherapy method” and “psychological well-being developer.” Participants recognized the VR psychotherapy method as an applicable approach capable of enhancing their psychological well-being.

- Applicable psychotherapy method

The participants stated that the VR program is so applicable for them. In this way, they expressed that the program was so helpful and useful for improving their mood and keeping them feeling relaxed.

*“The training was very helpful and practical for me, and it reduced my depression. I felt very relaxed” (P12).*

The participants stated that the VR positive psychotherapy method is an adjunctive psychotherapy method that can improve their psychological condition.

*“Your program, along with the medications I was taking, made me generally better. I mean, I felt much improved" (P13)*.

- Psychological well-being developer

The participants reported a development in their psychological well-being following the utilization of the VR positive psychology program. They highlighted enhancements in their spirit, positive feelings, and coping mechanisms. The participants also experienced a reduction in negativity post-intervention.

*“The fact that I was going next to nature or using things related to gratitude was very good for improving my mood” (P3).*

*“It was very good training for me. I had a lot of negative feelings, thought about suicide, and felt guilty. After receiving these teachings, I learned to love myself, not to care about the past, and to think about positive things. I also didn’t let my mind focus on negative things and suicide anymore” (P11).*

*“Before using this program, I was very aggressive and irritable. I used to cry very quickly, but now I’ve found myself, and I’m not like that anymore. The training taught me that nothing is more important than one’s health and peace of mind” (P2).*

Moreover, participants expressed an enhanced sense of self-connection. They remarked that they not only valued themselves but also nurtured self-love. They also stated that they accepted their past faults and guilt and had a profound capacity for self-forgiveness. Additionally, participants believed that they developed constructive cognitive self-awareness. They remarked on cultivating self-awareness of positive and negative thoughts, identifying self-empowerment, and reflecting on and remembering their blessings and strengths. Some participants stated that:

*“As a result of this method, I was able to forgive myself, appreciate myself, and love myself. I could see the beauty and goodness in my life, recognize my capabilities, and be grateful for them. Moreover, I could focus on the positive aspects of my life. Essentially, the awareness I gained about my positive and negative emotions helped me prevent negative thoughts from coming back to me” (P1).*

*“I always had problems with my family, and I didn’t see my own faults and mistakes, and I didn’t accept them. Now, I have accepted my faults and mistakes, and I look at the circumstances realistically and optimistically" (P10).*

## Discussion

In the present mixed-methods study, we examined whether two delivery formats of positive psychotherapy (VR-based and face-to-face) were associated with changes in well-being-related outcomes, including positive and negative affect, life satisfaction, and suicidal ideation, among individuals diagnosed with MDD.

Based on the results of the quantitative phase, the change in positive and negative affects, satisfaction with life, and suicidal ideation over time wasn’t significantly different between the groups. Improvements in positive and negative affect, life satisfaction, and suicidal ideation were observed over time in both groups. The subsequent qualitative study performed on the intervention group provided additional insights into participants’ experiences with the VR‑based intervention and potential factors influencing its perceived usefulness.

The magnitude of the improvements observed in the present study, reflected in small-to-moderate within-group effect sizes, appears broadly consistent with findings reported in the broader literature on positive psychology interventions (PPIs). Meta-analytic evidence indicates that PPIs generally yield small‑to‑moderate improvements in well-being and reductions in psychological distress. For example, Bolier et al. reported a pooled effect of approximately d = 0.34 for well‑being and d = −0.23 for depressive symptoms across randomized controlled trials of PPIs [25]. Similarly, Carr et al. found pooled effects of g = 0.39 for well‑being and g = −0.39 for depression across a large body of PPI studies [26]. A meta-analysis focusing specifically on depression also reported a pooled reduction in depressive symptoms of approximately d = −0.44 in pre‑post comparisons [23]. However, when PPIs are compared with other active psychological interventions, the differences appear smaller and often non-significant (e.g., Hedge’s g ≈ 0.15 for depression and g ≈ 0.20 for happiness) [28], suggesting that improvements associated with PPIs may be broadly comparable to those produced by other structured psychological approaches rather than uniquely large.

In the context of virtual-reality-based interventions, available evidence also points to modest but meaningful short-term improvements in affective states and well-being. For example, Habak et al. reported significant increases in well-being and positive affect, along with reductions in negative mood and hopelessness, following a brief immersive VR experience designed to enhance positive future thinking [38]. Likewise, a randomized crossover trial by Veling et al. demonstrated that VR-based relaxation significantly improved momentary affective states in psychiatric patients and produced greater reductions in negative affect than standard relaxation exercises, although no differences were observed for broader short-term symptom outcomes [37]. Taken together, these findings suggest that both traditional PPIs and VR-supported interventions tend to produce improvements that are generally small to moderate in magnitude, particularly in brief or short-term formats.

Against this background, the effect sizes observed in the present study fall within the range typically reported in the literature on positive psychotherapy and related VR-based interventions. Importantly, the comparable improvements observed across the face-to-face and VR delivery formats indicate that both modalities supported similar trajectories of positive change. These findings therefore point to equivalence in delivery format rather than differences in the efficacy of the intervention itself.

Based on the literature review, there aren’t any similar studies that compare the effect of VR positive psychotherapy intervention with in-person individual positive psychotherapy in patients with MDD. Therefore, we reviewed and discussed findings from previous studies examining the potential usefulness of these positive psychotherapy approaches among individuals with MDD. In the current study, participants receiving face‑to‑face positive psychotherapy showed improvements over time, including increases in positive affect and life satisfaction and decreases in negative affect and suicidal ideation. Consistent with the findings of the present study, previous research has also reported within-group improvements over time among individuals with MDD following in‑person positive psychotherapy [23,73–75]. For example, in the clinical trial conducted by Abdeyan et al. (2018), patients who completed eight 90‑minute sessions of group positive psychotherapy demonstrated significant increases in hopefulness across both post‑test assessments—immediately after the intervention and again two weeks later—indicating sustained improvement over time [74]. Similarly, Shaygan et al. (2022) reported time-related reductions in hopelessness and suicidal ideation among patients with MDD after eight sessions of group positive psychotherapy delivered alongside pharmacotherapy [73].

Overall, prior studies have reported improvements over time following in‑person positive psychotherapy among individuals with MDD, which is consistent with the within‑group improvements observed in the present study. Positive psychotherapy emphasizes helping individuals identify and cultivate personal strengths, abilities, and internal resources in a constructive manner. By focusing on these positive dimensions, PPT may enhance individuals’ sense of self‑empowerment and perceived control across multiple life domains, while also strengthening psychological resilience and adaptive coping with adverse experiences [76]. Through these mechanisms, interventions grounded in positive psychology may facilitate improvements in emotional functioning and well‑being by enhancing positive affect, fostering resilience, and encouraging engagement in meaningful and enjoyable activities. Moreover, in this study, participants in the face‑to‑face positive psychotherapy condition received individual sessions delivered over the intervention period. The observed improvements within this group may be related to participants’ greater engagement with the structured content of the sessions and their sustained participation across the intervention period. As a result, participants reported higher levels of positive affect and life satisfaction and lower levels of negative affect and suicidal ideation after the intervention compared with their baseline scores.

Patients receiving the VR‑based positive psychotherapy demonstrated overtime improvements in positive affect, negative affect, life satisfaction, and suicidal ideation. These findings are consistent with previous research indicating that VR interventions may support positive emotional experiences and improvements in psychological outcomes. Prior studies examining VR interventions for individuals with depressive symptoms have applied a range of VR-based approaches, including VR relaxation [37,77], VR behavioral activation [78], VR exposure therapy [79,80], VR‑based cognitive‑behavioral therapy [32,81,82], and VR‑based mindfulness programs [83]. Although recommendations exist to incorporate a wider range of strategies within VR interventions aimed at enhancing psychological outcomes [29] and promoting positive emotions among individuals with depressive symptoms [84], no research has yet explored the use of VR-based positive psychotherapy specifically for this population. Nevertheless, in two studies, some concepts related to positive psychotherapy were incorporated in a virtual therapy program to improve mental health in patients with fibromyalgia [85] and adults with a history of suicidality or self-identified depression [38]. For example, Herrero et al. (2014) found that an immersive VR environment designed to elicit positive emotions led to significant improvements in mood state, self‑efficacy, and motivation from pretest to posttest among patients with fibromyalgia [85]. Similarly, Habak et al. (2021) introduced a VR intervention designed to enhance future thinking, positive mood, and well‑being in patients with depression and prior suicidality. Following a single immersive VR session featuring diverse landscapes, participants showed significant increases in positive mood and well-being and reductions in hopelessness and negative mood from baseline to post-intervention [38]. Additionally, a study using a single-case experimental design showed that a VR intervention targeting positive autobiographical memories, as a way of inducing positive emotions, improved mood and enhanced strategies for regulating positive emotions in the short term among individuals with moderate-to-moderately severe depressive symptoms [86]. Several differences should be considered when comparing the present findings with the limited studies examining the use of positive psychotherapy delivered through VR, including differences in study populations, VR intervention methods and content, study designs, and research settings. Nevertheless, the existing evidence suggests the potential usefulness of VR-based psychological interventions, which is broadly consistent with the pattern of improvements observed over time in the present study.

In the qualitative phase of this study, participants recognized VR-based positive psychotherapy as an applicable and supportive approach that could enhance their psychological well-being. They reported that receiving positive-psychology-based guidance within a simulated natural environment improved their mood and fostered more positive feelings. Moreover, beyond the technological novelty of VR, its potential value may lie in its capacity to create immersive and emotionally engaging environments. Such environments can facilitate experiential learning and enhance participants’ emotional involvement with the intervention content, which may support the internalization of positive psychological concepts delivered during the intervention sessions. In addition, the VR-based positive psychotherapy program in this study was designed based on the literature on positive psychotherapy and Seligman’s theoretical framework to target well-being-related outcomes among individuals with MDD. In this regard, participants in the qualitative phase reported that the program addressed many of their key concerns and emotional challenges. Consistent with these perceptions, participants in the VR intervention showed improvements over time in emotional experiences, life satisfaction, and suicidal ideation. The findings indicated that there were no statistically significant differences between the two intervention formats in terms of emotional experiences (positive and negative affect), life satisfaction, or suicidal ideation. However, participants in both groups demonstrated improvements over time, including increased life satisfaction and positive affect as well as reductions in negative affect and suicidal ideation. Because both groups demonstrated similar improvements over time and no inactive control condition was included, the observed changes cannot be interpreted as definitive causal effects of either intervention format. Instead, the results indicate that the temporal pattern of change was comparable across the face-to-face and VR delivery modalities.

The study’s outcomes are aligned with Seligman’s theory, suggesting positive effects of positive psychotherapy interventions on emotional experiences and psychological well-being in patients with MDD. The PPT approach emphasizes the importance of focusing on individuals’ strengths, approaching challenges with a positive mindset, and fostering a belief in their ability to activate their potential. This approach helps patients address psychological and behavioral issues, ultimately guiding them toward better health and well-being [87]. In this method, individuals are encouraged to engage in mindfulness and self-awareness practices, which enhance their positive emotions and overall well-being [88,89]. Initially developed for those experiencing depressive symptoms [90], PPT has been proposed as a promising approach for enhancing psychological well-being among individuals with depressive symptoms.

Although no statistically significant differences were observed between the two intervention modalities, descriptive trends suggested that participants who received face-to-face positive psychotherapy reported slightly higher levels of positive affect and life satisfaction, along with lower levels of suicidal ideation across measurement points. These patterns may reflect some of the inherent advantages of in-person interactions, such as the opportunity to develop a stronger sense of connection with the facilitator, greater interpersonal engagement, and a more immediate sense of social presence. Consistent with this interpretation, participants in the qualitative phase emphasized the perceived importance of face-to-face psychotherapy, particularly during periods of psychological crisis.

At the same time, descriptive findings indicated that participants in the VR group showed a somewhat greater reduction in negative affect after the third session, although this difference did not reach statistical significance. One possible explanation is that certain elements of positive psychotherapy, such as exposure to natural environments, are difficult to implement within traditional clinical settings. The immersive natural environments provided through VR may offer patients an opportunity to experience restorative and emotionally engaging contexts that could help reduce negative affective states. In addition, VR environments may support emotional regulation through mechanisms such as attentional distraction, cognitive reframing, and the facilitation of positive imagery. These interpretations should, however, be considered tentative given the absence of statistically significant group differences.

Taken together, the findings of this study suggest that VR‑based delivery of positive psychotherapy may represent a feasible complementary format with outcomes comparable to those of traditional face‑to‑face therapy. Nevertheless, the present study should be considered preliminary. Future research with larger and more diverse samples, longer intervention periods, and extended follow‑up assessments is needed to further examine the potential usefulness of VR‑based positive psychotherapy and to clarify the conditions under which different delivery formats may be most beneficial. Additionally, future studies may explore whether integrating VR-based experiences with conventional face-to-face sessions could further enhance participant engagement and support improvements in positive psychological functioning.

Several limitations should be considered when interpreting the findings of this study. One important consideration is that participants may have been exposed to additional psychotherapy-related information through external sources such as practitioners, books, smartphones, or other media, which could have influenced the study outcomes. Although participants were instructed not to engage with other psychotherapy materials during the study period in order to minimize external influences, the possibility of such exposure cannot be completely ruled out. Participants were therefore encouraged to inform the researcher if they felt a need to seek additional positive psychotherapy resources during the study period.

Another limitation relates to the relatively short duration of the VR-based positive psychotherapy program, which may not have been sufficient to produce differential effects beyond those observed in the control condition. Additionally, the sample size was relatively modest, which may have limited the statistical power to detect between-group differences. Relatedly, the assessment of multiple well-being indicators in a relatively small sample introduces potential concerns regarding multiplicity; while the primary outcome was prespecified to mitigate this risk, findings related to secondary outcomes should be interpreted with appropriate caution and warrant replication in larger, adequately powered trials. Moreover, the use of face-to-face positive psychotherapy as an active control condition may also have reduced the likelihood of detecting additional benefits attributable to the VR-based intervention. Another methodological limitation is that the face-to-face psychotherapy sessions in the control group were delivered by a member of the research team (the second author). Although the sessions followed a standardized protocol and adherence was closely monitored, the involvement of a study author as the therapist may introduce the possibility of therapist-related bias. Future studies should consider using independent clinicians who are not part of the research team to further minimize potential bias. Furthermore, outcomes were assessed only in the short term, and the absence of longer follow-up assessments limits conclusions regarding the stability of the observed changes over time.

Another issue concerns the study’s predominantly female sample (over 90%), which may limit the generalizability of the findings to male populations. Another limitation relates to pharmacotherapy. Although all participants were prescribed sertraline as part of routine clinical care and dosages remained stable during the intervention, medication management was not an experimental variable in this study. As such, the potential background influence of ongoing pharmacotherapy cannot be fully excluded. Future research with larger and more gender-balanced samples, longer intervention and follow-up periods, and study designs that further account for the potential influence of concurrent pharmacotherapy may help to more clearly determine the specific contribution of VR-based positive psychotherapy.

## Conclusion

Based on the findings of this study, both face-to-face and VR-based positive psychotherapy were associated with improvements in positive and negative affect, life satisfaction, and suicidal ideation over time in individuals with MDD. The absence of significant between-group differences suggests that VR-based positive psychotherapy produced outcomes comparable to traditional face-to-face delivery in this preliminary trial.

Qualitative feedback indicated that participants generally perceived the VR program as user-friendly and engaging. These findings suggest that VR-based positive psychotherapy was perceived as feasible and acceptable within the present study, particularly among individuals who prefer technology-supported interventions.

Overall, these results provide preliminary evidence for the comparability of VR-based and face-to-face positive psychotherapy, highlighting VR as a promising modality for delivering positive psychotherapy. Further research with larger samples and longer follow-up is required to better understand its potential advantages and limitations.

## Supporting information

S1 File Kolmogorov–smirnov test results for assessing the normality of data distribution at baseline. (DOCX)

S2 Fig Individual trajectories of suicide ideation (B-SCS) across six time points in the intervention and control groups. (DOCX)

S3 Fig Individual trajectories of satisfaction with life (SWLS) across six time points in the intervention and control groups. (DOCX)

S4 Fig Individual trajectories of positive affect (PANAS) across six time points in the intervention and control groups. (DOCX)

S5 Fig Individual trajectories of negative affect (PANAS) across six time points in the intervention and control groups. (DOCX)
