# Supplementary material for: Assessing the effects of virtual reality-based positive psychotherapy on emotion, life satisfaction, and suicidal ideation in major depression: A mixed-methods randomized controlled trial
Source: PLoS One. 2026 Jul 30;21(7):e0354610. doi: 10.1371/journal.pone.0354610 (PMC13422861; doi:10.1371/journal.pone.0354610)
Supplement: S1 File — (DOCX) [file pone.0354610.s001.docx]

**Supporting information**

**S1 file. Kolmogorov–Smirnov test results for assessing the normality of data distribution at baseline.**

| **Variable** | **Group** | **Kolmogorov-Smirnov** | | | **Shapiro-Wilk** | | |
| --- | --- | --- | --- | --- | --- | --- | --- |
|  |  | **Statistic** | **df** | **Sig.** | **Statistic** | **df** | **Sig.** |
| **Negative affect** | **Intervention** | 0.131 | 39 | 0.09 | 0.951 | 39 | 0.09 |
|  | **Control** | 0.077 | 39 | 0.20^*^ | 0.971 | 39 | 0.39 |
| **Positive affect** | **Intervention** | 0.138 | 39 | 0.06 | 0.954 | 39 | 0.11 |
|  | **Control** | 0.132 | 39 | 0.08 | 0.944 | 39 | 0.053 |
| **Satisfaction with Life** | **Intervention** | 0.179 | 39 | 0.06 | 0.883 | 39 | 0.001 |
|  | **Control** | 0.128 | 39 | 0.11 | 0.932 | 39 | 0.02 |
| **Suicide ideation** | **Intervention** | 0.133 | 39 | 0.08 | 0.952 | 39 | 0.09 |
|  | **Control** | 0.107 | 39 | 0.20 | 0.965 | 39 | 0.26 |
