# Supplementary material for: Assessing the effects of virtual reality-based positive psychotherapy on emotion, life satisfaction, and suicidal ideation in major depression: A mixed-methods randomized controlled trial
Source: PLoS One. 2026 Jul 30;21(7):e0354610. doi: 10.1371/journal.pone.0354610 (PMC13422861; doi:10.1371/journal.pone.0354610)

**Supporting information**

**S2 fig. Individual trajectories of suicide ideation (B-SCS) across six time points in the intervention and control groups.**


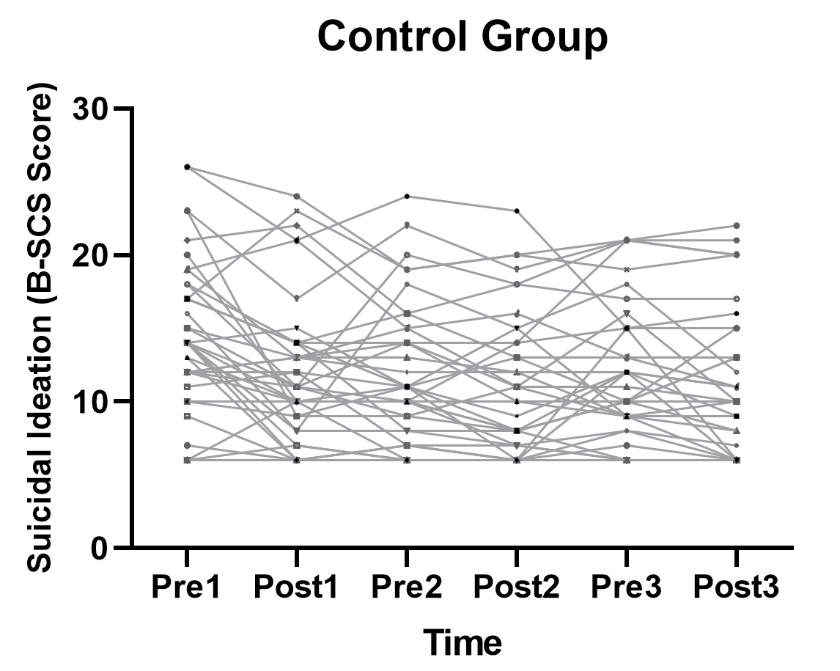


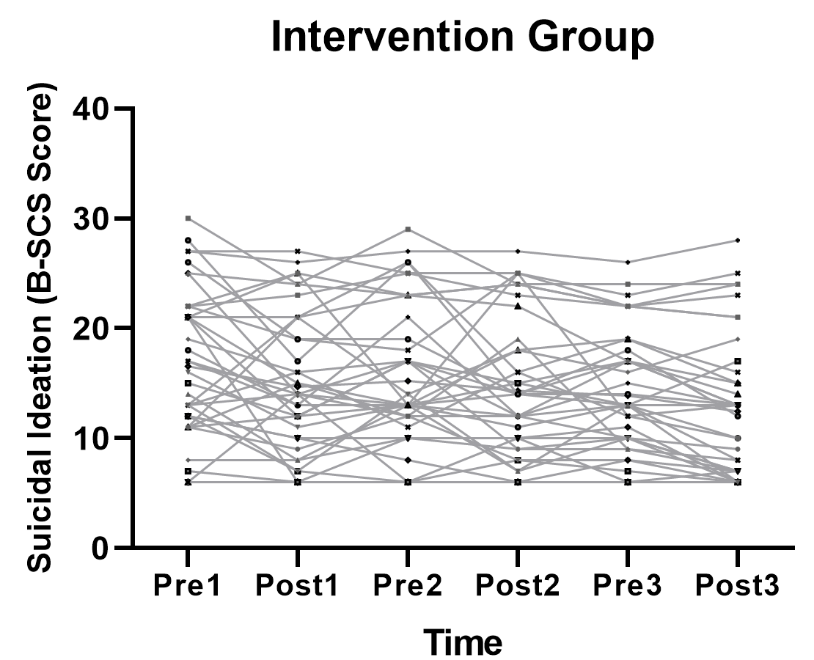

Supplement: S2 Fig — (DOCX) [file pone.0354610.s002.docx]
